# F-box protein FBXO31 is down-regulated in gastric cancer and negatively regulated by miR-17 and miR-20a

**DOI:** 10.18632/oncotarget.2183

**Published:** 2014-07-08

**Authors:** Xinchao Zhang, Ye Kong, Xia Xu, Huaixin Xing, Yingjie Zhang, Fengjuan Han, Wenjuan Li, Qing Yang, Jiping Zeng, Jihui Jia, Zhifang Liu

**Affiliations:** ^1^ Department of Biochemistry and Molecular Biology, School of Medicine, Shandong University, Jinan, P. R. China; ^2^ Department of Microbiology, Key Laboratory for Experimental Teratology of Chinese Ministry of Education, School of Medicine, Shandong University, Jinan, P. R. China; ^3^ Department of Anesthesiology, Shandong Cancer Hospital, Jinan, P.R. China; ^4^ Department of Radiation Oncology, Shandong Cancer Hospital; Jinan, P.R. China

**Keywords:** Gastric cancer, FBXO31, miR-20a, miR-17, CyclinD1

## Abstract

FBXO31, a subunit of the SCF ubiquitin ligase, played a crucial role in neuronal development, DNA damage response and tumorigenesis. Here, we investigated the expression and prognosis value of FBXO31 in human primary gastric cancer (GC) samples. Meanwhile, the biological role and the regulation mechanism of FBXO31 were evaluated. We found that FBXO31 mRNA and protein was decreased dramatically in the GC tissue compared with the adjacent non-cancerous tissues. FBXO31 expression was significantly associated with tumor size, tumor infiltration, clinical grade and patients' prognosis. FBXO31 overexpression significantly decreased colony formation and induced a G_1_-phase arrest and inhibited the expression of CyclinD1 protein in GC cells. Further evidence was obtained from knockdown of FBXO31. Ectopic expression of FBXO31 dramatically inhibited xenograft tumor growth in nude mice. miR-20a and miR-17 mimics inhibited, whereas the inhibitor of miR-20a and miR-17 increased, the expression of FBXO31, respectively. miR-20a and miR-17 directly bind to the 3'-UTR of FBXO31. The level of miR-20a and miR-17 in GC tissue was significantly higher than that in surrounding normal mucosa. Moreover, a highly significant negative correlation between miR-20a (miR-17) and FBXO31 was observed in these GC samples. Therefore, effective therapy targeting the miR-20a (miR-17)-FBXO31-CyclinD1 pathway may help control GC progression.

## INTRODUCTION

Gastric cancer (GC) is the fourth most common malignant tumor worldwide and ranks second in terms of cancer-related deaths [1,2]. According to the statistical data in 2008, the incidence rate of GC accounts for 8% of the total cancer and the mortality rate of GC accounts for 10% of total cancer-related deaths [1]. The highest incidence rate of GC is in Eastern Asia, especially in China [1,3]. Since it is difficult to be diagnosed in the early stage of this disease, most of the patients have reached the advanced or metastatic stage once be diagnosed. Conventional treatments with surgery, chemotherapies or radiation therapy play a minor role in improving the patients' survival rate [4,5]. Therefore, it is critical to find the new diagnostic or prognostic markers and therapeutic targets to improve the survival rate of GC patients.

FBXO31 belongs to F-box family. F-box containingproteins take part in the formation of Skp1-Cullin-F-box (SCF) ubiquitin E3 ligase complex, which mediates ubiquitin-dependent proteasome degradation pathway and regulates signal transduction, cell cycle progression, centrosome stability and mitotic fidelity [6-9]. F-box proteins have been classified into three family based on the presence of recognizable domains beyond the F-box domain. FBXW group harbors several WD40 repeats; FBXL group contained leucine-rich repeats and FBXO contain other domain. FBXO31 belongs to FBXO group [10,11].

FBXO31 located in chromosome 16q24.3 and played a crucial role in multiple pathways such as neuronal development [12],DNA damage response [13,14]and tumorigenesis [13,15,16, 17]. FBXO31 was initially considered as a candidate tumor suppressor since decreased FBXO31 expression was found in breast cancer. Ectopic expression of FBXO31 in breast cancer cells inhibited colony formation, cell proliferation and induced cellular senescence and G1 phase cell cycle arrest [15]. Decreased expression of FBXO31 was also found in hepatocellular carcinoma (HCC) [16]. However, opposite result was obtained in esophageal squamous cell carcinoma(ESCC). Kogo *et al* [17] showed that higher FBXO31 expression levels significantly correlated with elevated tumor invasion and clinical stage and determined poorer prognosis than the low expression group. The role of FBXO31 in tumorigenesis and development seems to be different in different tumor type. It is unclear the expression profile and function of FBXO31 in GC. Also it is incompletely understood how FBXO31 was regulated in cancer.

In the present study, we determined the expression level of FBXO31 in GC and the corresponding non-cancerous normal gastric mucosa tissues and analyze whether FBXO31 expression was associated with the clinicopathological variables and postoperative survival of GC patients. Then we detected the effect of FBXO31 on colony formation ability, cell-cycle distribution and the tumor formation ability *in vivo*. Finally, we investigated the molecular mechanism leading to the down-regulation of this gene in GC.

## RESULTS

### Decreased expression of FBXO31 mRNA and protein in primary GC tissues

**Figure 1:**
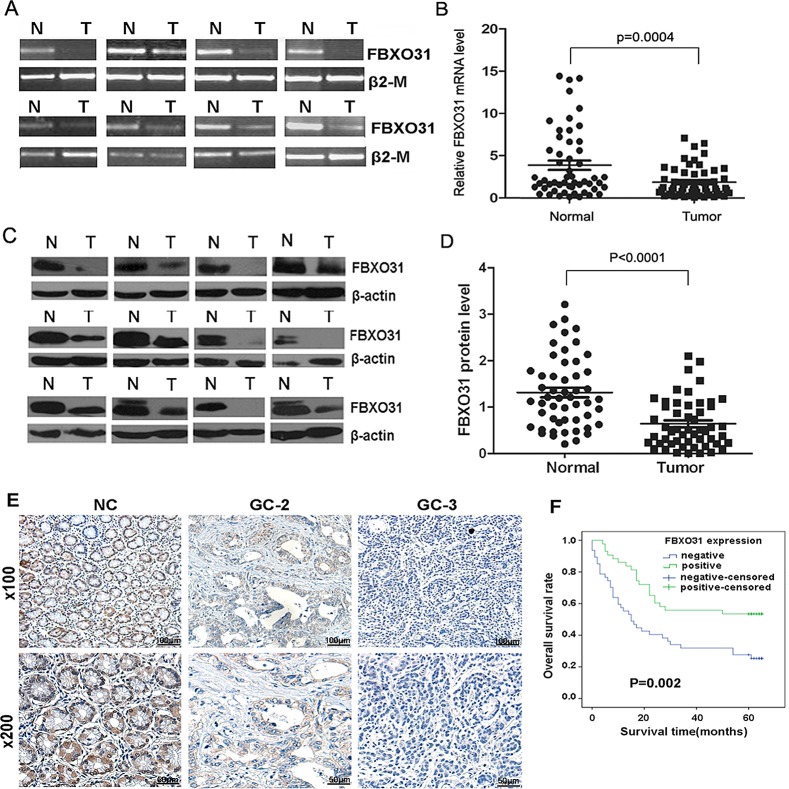
FBXO31 expression is decreased in human GC tissues and associated with clinical grade and patients' survival (A) RT-PCR analysis of FBXO31 mRNA level in GC specimens and adjacent non-cancerous tissues. Representative results were shown.β2-M was used as the control. N and T, normal and tumor tissue. (B) qRT-PCR analysis of FBXO31 mRNA expression levels in GC tissues and adjacent non-cancerous tissues (n=53, *P*=0.0004). Data, mean±SD. (C) Western blot analysis of FBXO31 protein level in GC and adjacent normal gastric mucosa. Representative results were shown. β-actin was used as the control. N and T: normal and tumor tissues. (D) Relative FBXO31 protein expression levels in GC tissues and non-cancerous tissues (FBXO31/β-actin, n=52, *P*<0.0001). Data, mean±SD. (E) Immunohistochemistry straining of the normal gastrictissues and the adjacent GC tissues. Representative results were shown. Strong FBXO31 staining was observed in non-cancerous gastric mucosa (NC). Weak FBXO31 staining was observed in GC with Grade 2(GC-2). Negative FBXO31 straining was found in GC with Grade3(GC-3).(F) Kaplan-Meier survival curves of GC patients (n=90) after gastrectomy. The survival rate of the patients in the FBXO31-positive group was significantly higher than that of patients in the FBXO31-negative group (log-rank test, *P*=0.002).

To investigate the role of FBXO31 in GC, we first evaluated the mRNA level of FBXO31 with RT-PCR and qRT-PCR in 53 paired GC tissues and the corresponding non-cancerous normal mucosa tissues. We found that the FBXO31 mRNA level was significantly lower in 38 (71.7%) GC tissues compared with the matched non-cancerous normal tissues (P=0.0004) (Fig.1A and 1B). As a control, the mRNA level of another F-box protein, FBXL10, has no significant differences between the GC tissues and the non-cancerous normal tissues (Fig.S1).

We further used western blotting to determine the protein level of FBXO31 in the paired 52 GC and corresponding non-cancerous normal tissues. The amount of FBXO31 protein was further measured by densitometry. A highly significant decrease in FBXO31 expression was found in 37 (71.2%) GC samples compared with their matched adjacent normal gastric tissues (p<0.0001) (Fig.1C and 1D), which is consistent with the qRT-PCR results. These data indicated that FBXO31 is significantly down-regulated at both mRNA and protein level in GC.

### FBXO31 expression level was associated the clinicopathological variables and postoperative survival of GC patients

To further investigate whether the expression level of FBXO31 was correlated the clinicopathological variables and patients' survival, we carried out immunohistochemical analyses of the tissue array including 90 paired paraffin-embedded GC and matched non-cancerous tissue. We found that the FBXO31 protein was readily detectable in all non-cancerous gastric tissues. However, 52.2% (47 of 90) of the GC tissue exhibited negative FBXO31 staining and 38.9% (35 of 90) showed weak FBXO31 staining (Fig. 1E). Overall, 84.4% of the GC tissue showed decreased expression of FBXO31 compared with the non-cancerous tissues, which further confirmed that the FBXO31 was down-regulated in GC tissue. The correlation between the expression of FBXO31 and various clinicopathological parameters are further analyzed. The data showed that the expression level of FBXO31 was significantly associated with tumor size (P=0.022), depth of tumor infiltration (T stage, P=0.024) and tumor grade (P=0.012), but not with age, gender, and tumor local lymph node metastasis (N stage) (Table 1). Univariate Cox regression analyses indicated that tumor size (P=0.016), depth of tumor infiltration (P=0.044), local lymph node metastasis (P<0.001) and FBXO31 expression (P=0.004) were significantly associated with patients' survival. Furthermore, a multivariate Cox regression analysis further confirmed the local lymph node metastasis (P=0.003) and FBXO31 expression (P=0.026) as independent predictors of the overall survival of GC patients (Table 2). The overall survival of patients with negative FBXO31 expression was significantly worse than that of FBXO31-positive patients (P = 0.002, log-rank test, Fig. 1F).

**Table 1:** Association between FBXO31 expression and clinicopathological variables of 90 gastric cancer cases

Clinicopathological parameters	n	FBXO31 expression		χ^2^	P value
		positive	negative		
all	90	43	47		
age(year)				1.558	0.212
<60	31	12	19		
≥60	59	31	28		
gender				0.03	0.863
male	62	30	32		
female	28	13	15		
tumor size(cm)				5.257	0.022^*^
≤5	41	25	16		
>5	49	18	31		
Tumor infiltration				7.442	0.024^*^
T1/T2	11	8	3		
T3	56	29	27		
T4	23	6	17		
Local lymph node metastasis				6.87	0.076
N0	25	14	11		
N1	10	8	2		
N2	26	10	16		
N3	29	11	18		
Distant metastasis				-	-
M0	89	42	47		
M1	1	1	0		
Grade				8.828	0.012^*^
1	16	13	3		
2	63	26	37		
3	11	4	7		

n Numbers of cases in each group.

* Statistically significant(P<0.05).

**Table 2:** Univariate and multivariate analyses of overall survival of gastric cancer patients

Variables	n	Univariate analyses			mulvariate analyses		
		HR	(95% CI)	P value	HR	(95% CI)	P value
age(year)				0.285			
<60	31	1.000					
≥60	59	1.367	0.771-2.422				
gender				0.356			
male	62	1.000					
female	28	1.296	0.747-2.246				
tumor size(cm)				0.016^*^			0.139
≤5	41	1.000			1.000		
>5	49	1.972	1.135-3.424		1.538	0.869-2.720	
Tumor infiltration				0.044^*^			0.810
T1/T2	11	1.000			1.000		
T3	56	2.985	0.917-9.718		1.546	0.414-5.766	
T4	23	4.52	1.322-15.446		1.541	0.368-6.455	
Local lymph node metastasis				<0.001^*^			0.003^*^
N0	25	1.000			1.000		
N1	10	3.851	1.171-12.663		5.166	1.506-17.726	
N2	26	5.734	2.142-15.351		4.138	1.437-11.915	
N3	29	7.957	3.012-21.023		6.509	2.389-17.739	
Distant metastasis				0.100			
M0	89	1.000					
M1	1	5.467	0.722-41.396				
Grade				0.192			
I/II	6	1.000					
III	63	1.967	0.885-4.383				
IV	11	1.293	0.435-3.849				
FBXO31				0.004^*^			0.026^*^
Negative	47	1.000			1.000		
Positive	43	0.440	0.254-0.764		0.500	0.271-0.919	

HR, hazard ratio;

CI, confidence interval;

n Numbers of cases in each group;

* Statistically significant(P<0.05).

### FBXO31 inhibits colony formation and induces G1 phase arrest in GC cells

**Figure 2:**
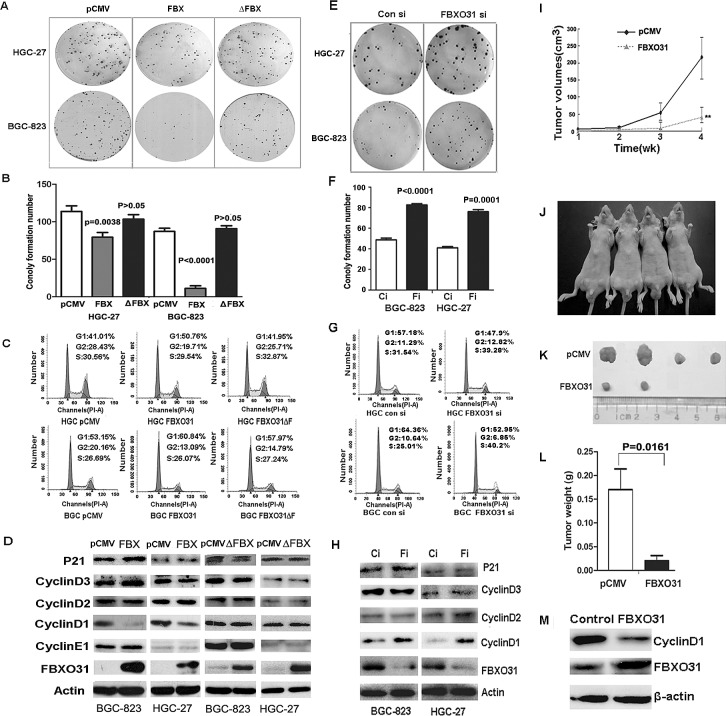
FBXO31 inhibited the colony formation and induced G1-phase cell cycle arrest *in vitro* and inhibited the tumor formation ability *in vivo* pCMV: the control vector; FBX: the vector expressing wild-type FBXO31; ΔFBX:the vector expressing mutant FBXO31.Ci:Control siRNA; Fi:FBXO31 siRNA.(A) The clonogenic potentials in the gastric cancer cells transfected with FBXO31 vector were assessed. The representative results were shown. (B)The colony formation number was analyzed in the cells transfected with different vector. Data, mean±SD. (C) The cell cycle distribution was analyzed by flow cytometry in the gastric cancer cells transfected with different vector. (D) Expression of the cell cycle regulators was determined with western blot. (E) The clonogenic potentials in the cells transfected with control siRNA and FBXO31 siRNA were assessed. The representative results were shown. (F) The colony formation number was analyzed in the gastric cancer cells transfected with control siRNA and FBXO31 siRNA. Data, mean±SD. (G) The cell cycle distribution was analyzed by flow cytometry in the gastric cancer cells transfected with control siRNA and FBXO31 siRNA. (H) Expression of the cell cycle regulators was determined with western blot in the cells transfected with control siRNA and FBXO31 siRNA. (I) The volumes of the subcutaneous tumors were measured weekly with a vernier caliper after implantation. (J)The nude mice were subcutaneously injected with BGC-823 cells transfected with pCMV (left) and FBXO31 (right) expression vector in the two flanks region. After 4 weeks, the nude mice were sacrificed.(K)The images of the dissected tumors are shown. A ruler is used to indicate the size of the tumor. (L)The tumor weight with different transfection was shown. Data, mean ±SD. (M) The total protein was extracted from the subcutaneous tumor tissues of the control groups and FBXO31 overexpression group. The protein levels of FBXO31 and cyclinD1 were determined with western blot. β-actin was used as the loading control.

Having obtained the results from the clinical data, we next investigated the biological function of this gene in GC cells. We transfected the control vector (pCMV-myc), the vectors expressing wild-type FBXO31 (pCMV-myc-FBXO31) and the F-box domain deletion mutation vector (pCMV-myc-FBXO31ΔF) into the GC cell lines BGC-823 and HGC-27, respectively. The ectopic expression of wild-type FBXO31 in the GC cells led to significant reduced foci numbers as well as sizes as compared with the control vector. But the transfection of F-box domain deletion vector has no this effect (Fig.2A and 2B). So, this inhibitory effect of FBXO31 on colony formation ability was dependent on its F-box domain. The cell cycle arrest is an important anti-proliferation mechanism in cancer [5,18, 19]. Therefore,we used flow cytometry to analyze the cell cycle and determine if the mechanism of the growth suppression of FBXO31 is due to the effect on the cell cycle. We found that the ectopic expression of wild type FBXO31 increases the cell population in G1 phase. But the ectopic expression of F-box deletion mutant FBXO31 (FBXO31ΔF) has no effect on the cell cycle distribution (Fig. 2C). Therefore, the F-box domain of FBXO31 is required for the the growth inhibition of FBXO31 on GC cells.

Then, we knocked down FBXO31 using FBXO31 specific siRNAs in the gastric cancer cell lines BGC-823 and HGC-27. Efficient inhibition of FBXO31 expression in the cells was verified using western blot analysis (Fig. 2H). The decreased expression of FBXO31caused a significant increase in foci numbers and sizes (Fig.2E and 2F). Furthermore, we used flow cytometry to analyze the cell cycle and found that FBXO31 inhibition decreased the cell numbers in G1 phase and promoted cell cycle progression (Fig.2G).

### FBXO31 inhibited the expression of CyclinD1 in GC cells

To further explore the underlying molecular mechanism leading to cell cycle arrest,we examined the expression of a series of cell cycle regulatory proteins in the GC cells, including P21,CyclinD1,CyclinD2,CyclinD3 and CyclinE1. Our result showed that ectopic expression of wild-type FBXO31 significantly inhibits the expression of cyclinD1,while the F-box domain deletion mutant FBXO31(FBXO31ΔF) has no effect on the expression of CyclinD1 (Fig.2D). FBXO31 inhibition by siRNA increased the expression of cyclinD1 significantly (Fig.2H). Moreover, FBXO31 overexpression or knockdown produced no significant changes in protein level of other regulator, including CyclinD2,CyclinD3,CyclinE1 and the inhibitor of CDK(CDKI; P21). These data indicate that FBXO31 induces G1 phase arrest in GC cells by inhibiting CyclinD1.

### FBXO31 impaired the tumorigenesis in nude mice

We then asked whether FBXO31 overexpression inhibited the tumorigenesis of GC cells *in vivo*. We thus subcutaneously injected BGC-823 cells transfected with FBXO31 overexpression vector or control vector into the nude mice and then examined their tumorigenic potential. After 1 week, the subcutaneous tumor size was measured every 7 days and the tumor volume was calculated. The result illustrated in Fig.2I showed that the tumor volume increased dramatically from pCMV-transfection group after 2 weeks. But the increase of the tumor volume in the FBXO31-transfection group was unapparent (Fig.2I). After four weeks, the mice were sacrificed. We found that xenografted tumor volume in FBXO31- transfected group was much smaller than that in pCMV-transfected group (Fig.2J and 2K). The tumor weight in pCMV-transfected group was significantly heavier than that in FBXO31-transfected group (P=0.0161; Fig.2L). The Cyclin D1 expression level was low in the tumor samples of FBXO31 overexpression group, which was similar with what we observed *in vitro* (Fig.2M). Especially, there is no tumor formation in two mice injected with FBXO31-transfected cells at the end of the observation period. Therefore, FBXO31 played a very important role in inhibiting tumor formation.

### FBXO31 was negatively regulated by miR-17 and 20a

**Figure 3:**
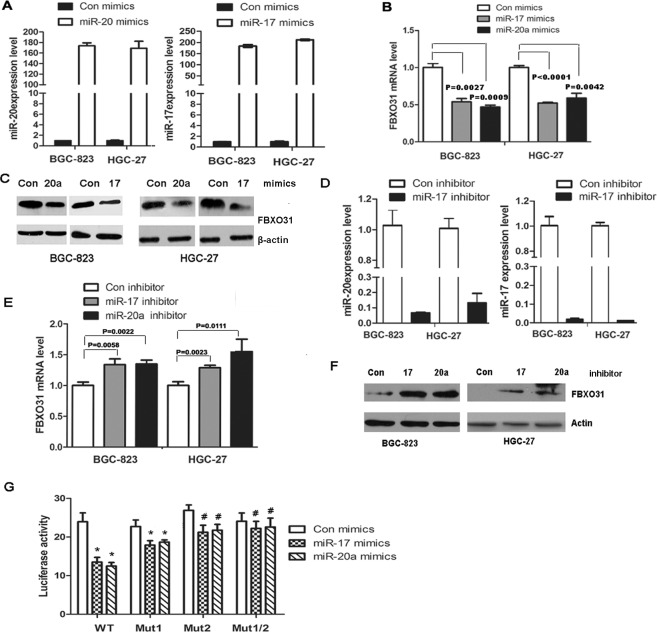
miR-20a and miR-17 directly bind to the 3′ untranslated region (UTR) of FBXO31 and inhibit FBXO31 mRNA and protein expression in human GC cells (A) miR-20a and miR-17 were analyzed with qRT-PCR in BGC-823 and HGC-27 cells transfected with miR-20a, miR-17 mimics or control mimics. The results confirmed the transfection efficiency. (B) The mRNA of FBXO31 was analyzed with qRT-PCR in BGC-823 and HGC-27 cells transfected with miR-20a or miR-17 mimics. (C) The protein of FBXO31 was analyzed with Western blot in BGC-823 and HGC-27 cells transfected with miR-20a or miR-17 mimics. (D) qRT-PCR analysis of the mRNA level of miR-20a and miR-17 in BGC-823 and HGC-27 cells transfected with miR-20a, miR-17 inhibitor or control inhibitor.(E) qRT-PCR analysis of the expression of FBXO31 in BGC-823 and HGC-27 cells transfected with miR-20a and miR-17 inhibitor.(F)Western blot analysis of the protein levels of FBXO31 in BGC-823 and HGC-27 cells transfected with miR-20a and miR-17 inhibitor.(G) The wild-type or mutant FBXO31 3'-UTR reporter constructs were co-transfected with miR-20a mimics, miR-17 mimics or control mimics into BGC-823 cells. Luciferase activity was determined at 48 hrs and was normalized to Renilla luciferase activity (*:P<0.05;#:P>0.05).

Since FBXO31 was markedly down-regulated in GC samples and played a very important role in inducing G1 phase arrest and inhibiting tumor formation *in vivo*, we next sought to explore the molecular mechanism leading to the down-regulation of FBXO31 in GC. Because miRNAs, a class of short non-coding RNAs consisting of 19-25 nucleotides, inhibit gene expression by binding to the complementary sequences of 3'-untranslated region (UTR) of target mRNAs and may lead to the inhibition of translation or degradation of the targeted mRNAs[20,21], we determined whether FBXO31 was negatively regulated by miRNAs. We used different databases, such as TargetScan, pictar and miRanda to predict the miRNA targeting FBXO31 and found that miR-20a and 17 were partly complementary to two conserved site within the 3' UTR of FBXO31. Therefore,we transfected the mimics or inhibitor of miR-20a or miR-17 into GC cells BGC-823 and HGC-27 and used qRT-PCR and western blot to detect the expression of FBXO31. We found that miR-20a or miR-17 mimics decreased, whereas miR-20a or miR-17 inhibitor increased, the mRNA and protein level of FBXO31, respectively (Fig.3 A-F). However, miR-20a and 17 mimics or inhibitor have no effect on the protein expression level of another F-box protein FBXL11 (Fig.S2).

To further determine whether FBXO31 was a direct target of miR-20a and miR-17,we constructed a vector containing the 3'UTR of FBXO31 and luciferase reporter vector pMIR-REPORT (pMIR-FBX) and investigated the effect of miR-20a and miR-17 on the luciferase activity of pMIR-FBX. We found miR-20a and 17 significantly reduced the luciferase activity of pMIR-FBX (Fig.3G). Because two sites within the 3'UTR of FBXO31 was found to be complementary to miR-20a and 17, we constructed three mutant, pMIR-FBX/mut1 (Site1 was mutated), pMIR-FBX/mut2 (Site2 was mutated) and pMIR-FBX/mut1,2 (Both site1 and 2 were mutated). Luciferase reporter assays showed that pMIR-FBX/mut1 exhibited significant inhibitory effect on luciferase activity, while pMIR-FBX/mut2 displayed a weak inhibitory effect and pMIR-FBX /mut1,2 showed completely reverse of the inhibitory effect on the luciferase activity compared with the wild-type vector (Fig.3G). Therefore, the second region of the 3' UTR of FBXO31 is important in binding with the miR-20a and miR-17.

### miR-20a and 17 were up-regulated in GC samples and FBXO31 expression was negatively associated with miR-20a and 17 in primary GC tissues

**Figure 4:**
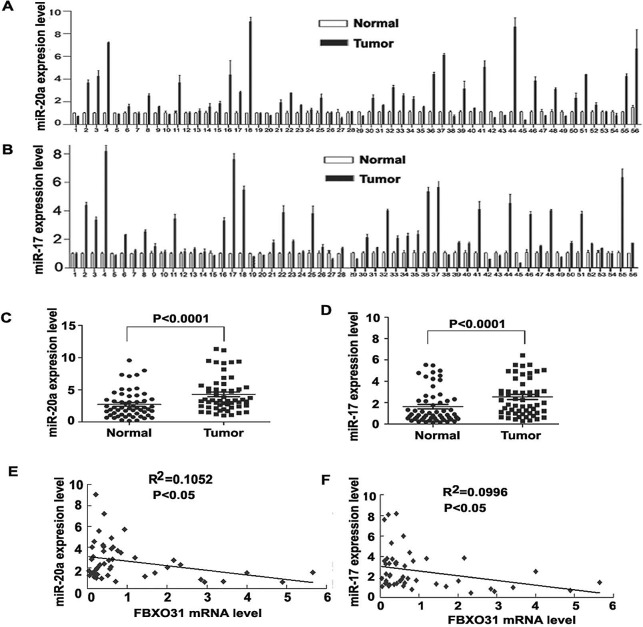
FBXO31 expression is negatively associated with miR-20a and miR-17 in primary GC tissues (A and B) qRT-PCR analysis of miR-20a (A) and miR-17 (B) level in 56 paired human GC and adjacent normal gastric mucosa tissues. Data are mean ± SD from three repeats. (C and D) Quantitative analysis of miR-20a(C) and miR-17(D) expression in GC tissues and adjacent normal gastric mucosa (n=56, P<0.0001). Horizontal lines represent the mean±SD. (E) Regression analysis of correlation of FBXO31 and miR-20a expression. (F) Regression analysis of correlation of FBXO31 and miR-17 expression. Each point represents one cancer sample.

Finally, we investigated whether miR-17 and 20a were up-regulated in primary GC tissues and associated with FBXO31. We used qRT-PCR to determine miR-20a and 17 expression in 56 paired GC and the corresponding non-cancerous normal mucosa tissues. In all, 37/56 (66.1%) and 38/56 (67.9%)of the clinical GC specimens showed increased expression of miR-20a and miR-17,respectively, as compared with surrounding normal mucosa (Fig.4A and 4B). Statistical analysis showed that the expression of miR-20a and 17 in tumor tissue was significantly higher than that in surrounding normal mucosa. (Fig. 4C and 4D). 26/52 samples showed the inverse trend of decreased FBXO31 and increased miR-20a expression in tumor tissues compared with non-cancerous tissue. 27/52 samples showed the inverse trend of decreased FBXO31 and increased miR-17 in tumor tissues compared with non-cancerous tissue. Statisticalanalysis showed that FBXO31 were highly correlated with miR-20a and miR-17 levels in GC samples (P<0.0001) (Fig. 4E and 4F).

## DISCUSSION

FBXO31 belongs to the human F-box family. F-box containing proteins exist as the component of SCF ubiquitin ligase complexes that are involved in ubiquitin-dependent proteasome degradation pathway [16]. The SCF complex includes the catalytic core complex consisting of Skp1, Cullin1, and the E2 ubiquitin-conjugating (Ubc) enzyme [22,23] and the F-box protein that targets hundreds of substrates through phosphospecific domain interactions [11]. Some F-box proteins, such as SKP2, display oncogenic function, while other proteins, such as FBXW7,act as tumor suppressors [24-31]. FBXO31 was initially regarded as a candidate tumor suppressor [15]. The expression level of FBXO31 is relatively lower in the breast cancer and hepatocellular carcinoma tissues compared with their corresponding normal tissues [15,16]. However, a recent study from Kogo et al. showed a conflicting result in esophageal squamous cell carcinoma (ESCC) [17]. They found that the expression level of FBXO31 was positively associated with tumor invasion depth and clinical stage. Moreover, higher FBXO31 expression correlated with poorer prognosis in ESCC patients. Therefore, the expression and function of FBXO31 is controversial in different cell type. It is unclear about the expression profile, clinical significance, biological functions and the regulation mechanism of FBXO31 in GC. In this study, we investigated the expression level of FBXO31 in GC and corresponding non-cancerous tissues and analyzed the clinicopathological and prognostic significance in these human samples. We further evaluated the biological function of FBXO31 in GC cells and nude mice xenograft model. Finally, we explore the molecular mechanism of FBXO31 down-regulation in GC.

In the present study, our qRT-PCR and western blot results showed that both the mRNA and protein levels of FBXO31 was significantly decreased in GC tissues compared with corresponding non-cancerous mucosa. Consistent with these findings, immunohistochemical results in the 90 paired paraffin-embedded gastric tissue array indicated that FBXO31 was completely silenced in 47 out of 90 patient GC samples, with weak positive expression in another 35 patients. Further analysis showed that the decreased expression of FBXO31 was significantly associated with a higher clinical stage of GC, suggesting that FBXO31 might play an important role in tumor development and progression. Furthermore, a survival analysis indicated that the loss or decreased expression of FBXO31 was significantly associated with low survival rate and poor prognosis, implying that it may serve as a valuable prognostic marker and a potential therapeutic target for GC patients. Our result indicated that FBXO31 was a candidate tumor suppressor in GC, which is consistent with the results in breast cancer and hepatocellular carcinoma.

The results from the breast cancer and hepatocellular carcinoma indicated that ectopic expression of FBXO31 inhibited cell growth and blocks cells at G0-G1 phase of the cell cycle [15,16]. In our study, restoring the expression of wild-type FBXO31 in GC cells also significantly inhibited the colony formation and led to G1 phase cell cycle arrest, while the F-box domain mutant FBXO31ΔF abolished the inhibitory ability. Therefore, the F-box domain is required for the biological function of FBXO31. Santra et al. showed that the F-box motif of FBXO31 interacts with cyclinD1, an important regulator of G1 to S phase progression, and mediates the degradation of Cyclin D1 through a ubiquitin-proteasome pathway [13]. Therefore, we detected the expression of cyclinD1 and other cell cycle regulator CyclinD2,CyclinD3,CyclinE1 and P21 in the GC cells transfected with FBXO31 expression vector or FBXO31 siRNA and found that FBXO31 overexpression suppressed, whereas its inhibition increased, the expression of CyclinD1,respectively. In order to further verify the growth inhibitory role of FBXO31 *in vivo*, we performed the nude mice xenograft experiment. In our experiments, overexpression of wild-type FBXO31 significantly inhibited the tumorgenesis in nude mice. These data suggest that FBXO31 plays a tumor suppressor role in tumor formation.

Currently, little is known about how FBXO31 expression is regulated. Accumulating evidences highlight the importance of miRNAs in regulating gene expression and involving in tumorigenesis and progression. MiRNAs can interact with the complementary sequences in the 3′UTR of the target mRNA to induce translational repression or target degradation [20,21]. Some members of F-box family can be regulated by miRNAs. FBXW7 was regulated by miR-223 and miR-27a and served as a tumor suppressor to inhibit tumor growth and progression [32-36]. FBXW11 was regulated by miR-106b-25 cluster and involved in tumor invasion and metastasis [37]. SKP2 was regulated by miR-7 [38]. We then asked whether FBXO31 was regulated by miRNAs. We used different databases, such as TargetScan, pictar and miR and a to predict the miRNA targeting FBXO31 and found that miR-17-92 cluster were complementary to the 3' UTR site of FBXO31. Furthermore,the two members of miR-17-92 cluster, miR-17 and 20a, are important markers for GC [39,40]. Therefore, we detected whether FBXO31 were regulated by miR-17 and miR-20a. Our results indicated that miR-17 and miR-20a mimics inhibited, whereas miR-17 and miR-20a inhibitor increased, the expression of FBXO31. Clinically,we found that the expression of miR-17 and miR-20a in tumor tissue was significantly higher than that in surrounding normal mucosa. A highly significant negative correlation between miR-17 (20a) and FBXO31 was observed in these GC samples. Therefore, the increased miR-17(20a) expression in GC tissue contributed to the down-regulation of FBXO31 partly. It is needed to be further investigated whether other mechanism, such as DNA methylation, loss of heterozygosity(LOH) contributed to the down-regulation in GC tissue.

Collectively, our data demonstrated that FBXO31,a member of F-box family, exerts tumor-suppressive function in GC. Loss of FBXO31 expression in GC correlated with a more malignant phenotype and poorer prognosis in GC. FBXO31 induces G1 phase cell cycle arrest and causes the growth inhibitory role of GC both *in vitro* and *in vivo*. The overexpression of miR-17 and miR-20a contributed to the down-regulation of FBXO31 in GC tissues partly. These results suggest miR-17(20a)-FBXO31-CyclinD1 pathway may be a potential therapeutic target of GC.

## MATERIALS AND METHODS

### Cell lines, siRNA, miRNAs and plasmids

Human GC cell lines BGC-823, HGC-27 (purchased from the Cell Resource Center, Institute of Biochemistry and Cell Biology at the Chinese Academy of Sciences, Shanghai, PR China) were used in the present study. Cells were cultured at 37°C, 95% air, 5% CO_2_ in RPMI 1640 medium (Invitrogen, Carlsbad, CA) containing 10% fetal bovine serum (FBS),100 U/mL penicillin and 2 mmol/L L-glutamine. The sequences for the FBXO31 siRNA were: 5'-CUGAUGAAGUUCAUCUACAUU-3' [13]and were synthesized from GenePharma. Human miR-20a mimics, miR-17 mimics, control mimics, miR-20a inhibitors, miR-17 inhibitors and control inhibitors were synthesized from RiBoBio (Guangzhou, China). pCMV-myc-FBXO31 vector and F-box domain deletion mutation vector pCMV-myc-FBXO31ΔF were kindly provided by Prof. David F. Callen (Dame Roma Mitchell Cancer Research Laboratories, Department of Medicine, University of Adelaide and Hanson Institute).

### Cell transfection

FuGENE HD Transfection Reagent (Roche Applied Science) was used for transfection of pCMV-myc, pCMV-myc-FBXO31 or pCMV-myc-FBXO31ΔF plasmid into BGC-823 or HGC-27 cells. Lipofectamine 2000 (Invitrogen) was used to transfect miRNA mimics, inhibitor or siRNA into BGC-823 or HGC-27 cells. All transfection procedures followed the protocol of the manufacturer.

### RNA extraction, reverse transcription and qRT-PCR

TRIzol reagent (Invitrogen) was used to extract total RNA from the cells and tissue specimens. Primers for miR-20a, miR-17 and U6 were synthesized from RiBoBio (Guangzhou, China). The PCR primers for FBXO31 were synthesized from Invitrogen. The sequences for FBXO31 were as follows: 5'-CCGGCGGGAGGCAGGAGGAGT-3' (forward) and 5'-GCGGCGGTAGGTCAGGCAGTTGTCG-3' (reverse); for FBXL10: 5'-GTCTGATGAGCGTGAAAGGTTGT (forward) and 5'-TCCGCCGAACACCAAAGAGT-3' (reverse). The above primers cross intron/exon boundaries in the FBXO31 gene; thus, the resultant PCR products do not represent genomic DNA contamination. β2-microglobulin expression (β2-M) was used as a control for RNA loading and reverse transcription efficiency and amplified. The first-strand cDNA was synthesized with random primers (N6) or miRNA specific primers and M-MLV reverse transcriptase (Ferments). The quantitative real-time PCR (qRT-PCR) was performed using the Bio-Rad CFX96^TM^ Real-Time PCR System (Bio-Rad) with the SYBR Green Kit (TaKaRa) according to the manufacturer's instructions. Calculation of target mRNA levels was based on the CT method and normalization to human β2-M or U6 expression. All reactions were run in triplicate.

### Reporter vector construction and luciferase assay

The 217bp 3' -UTR sequence of human FBXO31 gene containing miR-20a and miR-17 binding sites was amplified and inserted into the SpeI/HindIII sites of the pMIR-REPORT luciferase vector (named as pMIR-FBXO31/wt). Two miR-20a and miR-17 complementary sequences GCACTTT in the 3' UTR were mutated singly or together to remove complementarity by use of a QuikChange site-directed mutagenesis kit with pMIR-FBXO31/wt as the template. All the primer sequences were listed in Table S1. The mutants were named pMIR-FBXO31/mut1, pMIR-FBXO31/ mut2 and pMIR-FBXO31/ mut1,2. GC cells were seeded in 24-well plates and transiently transfected with appropriate reporter plasmid and miRNA by use of Lipofectamine 2000. After 48 h, the cells were harvested and lysed. Luciferase activity was measured by use of the Dual-Luciferase Reporter Assay System (Promega, Madison, WI, USA). Renilla luciferase was used for normalization. For each plasmid construct, the transfection experiments were performed in triplicate.

### Western blot analysis

Total proteins from the cells or tissues were extracted with RIPA lysis buffer. Protein concentrations were measured by use of the BCA reagent kit (Merck). The proteins were resolved by SDS-PAGE and transferred to a PVDF membrane, which was probed with specific primary antibodies against FBXL11 (Abcam), FBXO31 (Abcam), CyclinE1(Cell Signaling Technology), P21 (Epitomics), CyclinD1 (Epitomics),CyclinD2 (Santa cruz biotechnology),CyclinD3 (Santa cruz biotechnology) followed by anti-mouse or rabbit horseradish peroxidase-conjugated IgG (Bio-Rad) and developed with the chemiluminescence method (ECL, Millpore). β-actin (Sigma-Aldrich) servedas a loading control.

### Colony formation assay

The treated cells were seeded into 6 well-plates (300 cells per well) and incubated for 10 days. Thereafter, the cells in the plates were fixed with methanol and stained with crystal violet and the number of colonies with more than 50 cells was counted. The experiments was performed in triplicate and repeated three times.

### Cell-cycle analysis

The transfected cells were harvested, fixed with 70% ethanol at 4°C for more than 4h and stained with propidium iodide (PI) containing RNase A at 37°C for 30 minutes in the dark. Cell-cycle distribution was determined using a flow cytometer (BD Biosciences). Each experiment was carried out in triplicate and the data were analyze dwith FCS Express V3.0612 soft.

### Nude mice xenograft model

Athymic BALB/c nude mice (5-6 weeks) were purchased from Peking University (Beijing, China) and bred under specific pathogen-free conditions. BGC-823 GC cells transfected with pCMV-myc or pCMV-myc-FBXO31 (5×10^5^ viable cells in 0.1 mL PBS) were subcutaneously injected into the two flank region of each mouse. After 1 week, the subcutaneous tumor size was measured every 7 days with a vernier caliper and the tumor volume was calculated by the formula (length)×(width^2^)/2 [5]. The mice were sacrificed after 4 weeks and the dissected tumors were collected and weighed. All the animal experiments were approved by the local ethics committee of Shandong university.

### Patients

We obtained fresh tumor specimens and surrounding normal tissue from 56 patients with primary GC who underwent gastrectomy at the Cancer Hospital of Shandong Province in 2012-2013. Samples were stored at -80°C. We collected data on patient age,sex,tumor histology,differentiation status, size (diameter), invasiveness, and regional and distant metastases at the time of surgery (pathologic tumor-node-metastasis classification). Detailed patient and disease characteristics are documented in Table S2. The study was approved by the ethics committee of School of Medicine, Shandong University.

### Tissue array

Commercially available paraffin-embedded human gastric tissue array was bought from Outdo (Shanghai Outdo Biotech Co.,Itd. Shanghai, China). One tissue array contained 180 spots,including 90 paired GC tissue and corresponding non-cancerous mucosa tissue. The data about the patients' gender, age, tumor size, clinicopathological and survival parameters were provided by the manufacturer.

### Statistical analysis

FBXO31 mRNA and protein expression differences between tumor tissues and matched non-tumor tissues were analyzed with the Student's *t*-test. The association between FBXO31 expression and various clinicopathological parameter was evaluated with the x^2^test. The Cox proportional hazard regression model was used for univariate and multivariate analyses to determine the effects of the clinicopathological variables and FBXO31 expression on the patients' survival. Survival curves were calculated using the Kaplan–Meier method. The differences of colony formation were assessed with the two-tailed unpaired Student's t test. Correlation analyses between FBXO31 and miR-20a (17) expression in GC samples were made using linear regression. Statistical analyses were performed with the Statistical Package for the Social Sciences, version 17.0 (SPSS Inc., Chicago, IL, USA),P<0.05 was considered statistically significant.

## SUPPLEMENTARY FIGURES AND TABLES


