# Supplementary material for: F-box protein FBXO31 is down-regulated in gastric cancer and negatively regulated by miR-17 and miR-20a
Source: Oncotarget. 2014 Jul 8;5(15):6178–90. doi: 10.18632/oncotarget.2183 (PMC4171621; doi:10.18632/oncotarget.2183)
Supplement: Supplementary file 1 [file oncotarget-05-6178-s001.pdf]

F-box protein FBXO31 is down-regulated in gastric cancer and negatively regulated by miR-17 and miR-20a

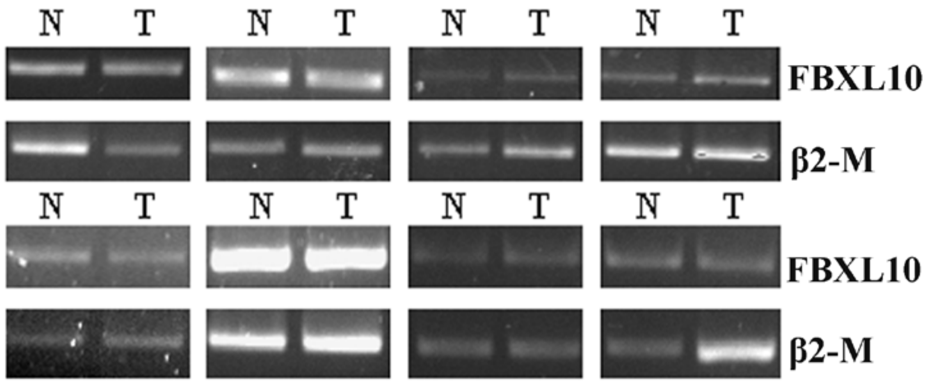

Figure S1.RT-PCR analysis of FBXL10 mRNA level in GC specimens and adjacent non-tumor tissues. Representative results were shown.β2-M was used as the control.N and T,normal and tumor tissue.

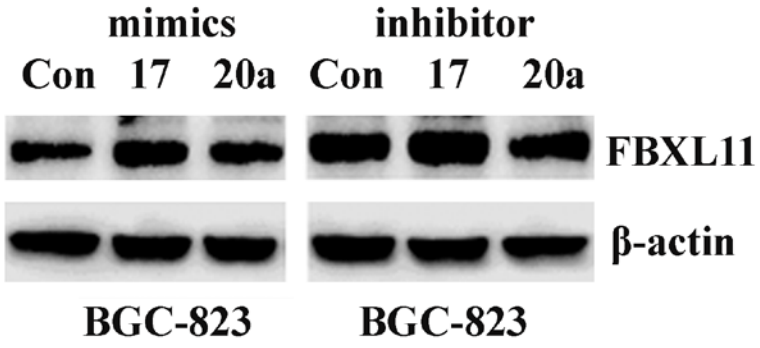

Figure S2.The protein of FBXL11 was analyzed with western blot in gastric cancer cells transfected with miR-20a and miR-17 mimics or inhibitor.β-actin was used as the loading control.

Table S1: Sequences for construction of wild type (pMIR-FBXO31/wt) and mutants (pMIR-FBXO31/mut1 and pMIR-FBXO31/mut2) of the 3’ UTR of FBXO31

Category	Sequences
Sequences for construction<br>of pMIR-FBXO31/wt	F:5’-GGACTAGTGGGGCTCTGAACTCTGACCT-3’<br>R:5’-CCCAAGCTTTCTGCTGTATTCAGGCTCGT-3’
Sequences for construction<br>of pMIR- FBXO31/mut1	F1:5’-GGACTAGTGGGGCTCTGAACTCTGACCTGTGAATAGAAGCAGCATCGTGATAGGAAATC-3’<br>R1:5’-GATTTCCTATCACGATGCTGCTTCTATTCACAGGTCAGAGTTCAGAGCCCCACTAGTCC-3’<br>**F2:5’-**GCAGCATCGTGATAGGAAATC -3’<br>R2:5’-CCCAAGCTTTCTGCTGTATTCAGGCTCGT-3’
Sequences for construction<br>of pMIR- FBXO31/mut2	F1:5’-CCCCCCACGTGATAATGTAGA-3’<br>R1:5’-CCCAAGCTTTCTGCTGTATTCAGGCTCGT-3’<br>F2:5’-GGACTAGTGGGGCTCTGAACTCTGACCT-3’<br>R2:5’-TCTACATTATCACGTGGGGGG-3’

Table S2. Patient and tumor characteristics, FBXO31 protein expression in normal and gastric cancer specimens

Patient and tumor characteristics					FBXO31 protein expression[3]	
No.	Age(yr)/Sex	Size(cm)	pTNM[1]	Histol/Dif[2]	Normal	Tumor
1	66/M	9.0	T4N3M0	MucinousA	0.66	0.31
2	64/F	3.5	T4N2M0	LDA	1.35	0.36
3	68/M	7.0	T4N3M1	MDA	1.58	0.38
4	51/M	3.0	T3N2M0	LDA	1.12	1.09
5	72/M	2.5	T4N3M0	LDA	1.32	0.89
6	63/M	5.5	T4N3M0	LDA	1.96	0.79
7	70/F	4.0	T4N1M1	MDA	1.67	0.10
8	62/M	5.0	T4N1M0	MDA	1.19	0.01
9	62/M	6.0	T4N2M0	LDA	0.43	0.03
10	58/M	2.0	T4N1M0	MDA	1.66	0.48
11	74/M	6.0	T3N0M0	MDA	1.98	1.18
12	50/F	4.0	T4N2M0	LDA	2.61	1.11
13	56/F	7.5	T4N1M0	LDA	0.20	0.56
14	62/F	3.5	T4N2M0	LDA	2.23	0.52
15	38/F	8.0	T4N1M0	LDA	1.09	1.33
16	59/M	4.0	T4N0M0	MucinousA	0.96	0.87
17	62/M	6.0	T4N3M0	LDA	1.78	0.01
18	58/F	4.0	T4N3M1	LDA	1.08	1.09
19	54/M	7.0	T4N0M0	MucinousA	0.75	0.23
20	31/M	6.0	T4N3M1	LDA	0.72	1.04
21	54/M	4.0	T2N0M0	LDA	2.12	0.41
22	61/M	12	T4N1M0	LDA	0.54	0.02
23	49/M	3.0	T4N0M0	LDA	0.82	0.14
24	40/M	6.0	T4N1M0	MDA	0.27	0.10
25	61/M	5.0	T4N0M0	LDA	0.38	0.15
26	37/M	6.0	T4N3M0	LDA	2.40	2.10

27	60/M	5.0	T4N3M0	LDA	0.45	0.73
28	49/F	3.0	T4N1M0	LDA	2.89	1.38
29	46/F	2.0	T4N2M1	LDA	1.17	0.13
30	59/M	6.0	T4N3M0	LDA	1.21	1.19
31	59/F	11	T4N3M0	LDA	0.98	0.24
32	64/M	5.5	T4N3M0	LDA	2.13	0.51
33	65/M	5.5	T4N3M0	MucinousA	1.06	0.22
34	68/M	4.5	T4N3M0	LDA	1.67	0.21
35	68/M	5	T4N3M0	LDA	-	-
36	52/F	9.0	T4N1M0	MDA	0.42	0.21
37	69/M	7.5	T4N3M1	LDA	1.35	0.47
38	46/F	2.0	T4N1M0	LDA	2.78	1.12
39	59/M	6.2	T3N0M0	LDA	2.69	1.02
40	68/M	7.0	T4N1M0	MucinousA	1.02	1.57
41	64/M	5.5	T4N1M0	LDA	0.57	0.27
42	69/M	8.0	T4N2M0	MDA	2.38	0.42
43	45/M	10	T4N2M0	LDA	1.21	1.98
44	65/M	10	T4N3M1	LDA	0.46	0.32
45	69/M	7.5	T4N3M1	LDA	0.51	0.57
46	75/M	9.5	T4N0MO	MDA	-	-
47	73/M	6.0	T4N2M0	LDA	0.48	0.26
48	77/M	1.5	T4N2M0	LDA	-	-
49	52/M	1.5	T4N3M0	LDA	-	-
50	37/F	13	T4N3M1	LDA	3.21	0.51
51	66/F	3.5	T4N2M0	LDA	0.88	0.59
52	30/F	4.5	T4N3M0	LDA	0.72	1.01
53	80/M	11	T4N3M1	MDA	1.76	1.81
54	56/F	5.4	T4N2M0	LDA	0.62	0.73
55	79/M	5.5	T4N2M0	MDA	1.42	0.09
56	56/M	5.5	T4N1M0	MDA	1.39	0.31

1.Pathologic tumor-node-metastasis.

2.Histology/Differentiation status: LDA, Low differentiated adenocarcinoma; MDA, Moderately differentiated adenocarcinoma; MucinousA, Mucinous adenocarcinoma.

3. FBXO31 expression levels in gastric cancer and matched non-cancerous specimens were analyzed using western blot. Relative abundances of protein were calculated based on the band density values and normalized with β-actin. -: undetectable.
